# Silver Nanoparticle-Based Therapy: Can It Be Useful to Combat Multi-Drug Resistant Bacteria?

**DOI:** 10.3390/antibiotics11091205

**Published:** 2022-09-06

**Authors:** Eva M. Mateo, Misericordia Jiménez

**Affiliations:** 1 Department of Microbiology and Ecology, Faculty of Medicine and Odontology, Universitat de Valencia, E-46010 Valencia, Spain; 2 Department of Microbiology and Ecology, Faculty of Biological Sciences, Universitat de Valencia, E-46100 Valencia, Spain

**Keywords:** *Acinetobacter baumannii*, antibiotics, cytotoxicity, Enterobacteriaceae, antibiotic resistant bacteria, nanomaterials, nanotherapy, *Pseudomonas aeruginosa*, silver, *Staphylococcus aureus*

## Abstract

The present review focuses on the potential use of silver nanoparticles in the therapy of diseases caused by antibiotic-resistant bacteria. Such bacteria are known as “superbugs”, and the most concerning species are *Acinetobacter baumannii*, *Pseudomonas aeruginosa*, *Staphylococcus aureus* (methicillin and vancomycin-resistant), and some Enterobacteriaceae. According to the World Health Organization (WHO), there is an urgent need for new treatments against these “superbugs”. One of the possible approaches in the treatment of these species is the use of antibacterial nanoparticles. After a short overview of nanoparticle usage, mechanisms of action, and methods of synthesis of nanoparticles, emphasis has been placed on the use of silver nanoparticles (AgNPs) to combat the most relevant emerging resistant bacteria. The toxicological aspects of the AgNPs, both *in vitro* using cell cultures and *in vivo* have been reviewed. It was found that toxic activity of AgNPs is dependent on dose, size, shape, and electrical charge. The mechanism of action of AgNPs involves interactions at various levels such as plasma membrane, DNA replication, inactivation of protein/enzymes necessary, and formation of reactive oxygen species (ROS) leading to cell death. Researchers do not always agree in their conclusions on the topic and more work is needed in this field before AgNPs can be effectively applied in clinical therapy to combat multi-drug resistant bacteria.

## 1. Introduction

The World Health Organization (WHO) has published a list of the emerging bacteria or bacterial families that pose the greatest threat to human health because they are resistant to many antibiotics and for which there is a very urgent need for new treatments [1,2,3,4]. The list ranks *Acinetobacter baumannii*, *Pseudomonas aeruginosa* and Enterobacteriaceae *(Klebsiella pneumoniae*, *Escherichia coli*, *Enterobacter* spp., *Serratia* spp., *Proteus* spp., *Providencia* spp., and *Morganella* spp.) extended-spectrum-β-lactamase-producing (carbapenem-resistant), followed by *Enterococcus faecium* (vancomycin-resistant), *Staphylococcus aureus* (methicillin, vancomycin resistant)*, Helicobacter pylori* (clarithromycin-resistant), *Campylobacter* spp. and Salmonellae (fluoroquinolone-resistant), *Neisseria gonorrhoeae* (cephalosporin, fluoroquinolone-resistant), *Streptococcus pneumoniae* (penicillin-non-susceptible), *Haemophilus influenzae* (ampicillin-resistant) and *Shigella* spp. (fluoroquinolone-resistant) as priority targets. Mycobacteria species were not included in this review to narrow down the scope of the present work and focus entirely on the main species listed by the WHO [1,2]. In the last years, the interest in nanotechnology has become increasingly important for global industries. Applications in medicine extend from the use of nanomaterials for medical devices to the use of nanoparticles (NPs) as therapeutic agents, drug delivery systems, or diagnostic imaging systems. NPs are engineered structures defined as particles with a diameter of 1–100 nm [5,6], though some of the reported NPs exhibit a size >100 nm [7]. According to Mitchell et al. [8], NPs can be classified as lipid-based, polymeric, and inorganic NPs; to which carbon-based NPs can be added [9]. Lipid-based NPs are usually spherical platforms comprising at least one lipid bilayer surrounding one or more internal aqueous compartments and are used as a delivery system. They include liposomes, lipid NPs, and oil/water emulsions. Their advantages include high bioavailability, formulation simplicity, or self-assembly, making them very useful in nanomedicine. Polymeric NPs include polymersomes, dendrimers, polymer micelles, and nanospheres. They are good delivery vehicles because of their biocompatibility and simple formulation parameters; they are hydrosoluble, stable, and well suited for the delivery of drugs encompassing different sizes, structures, and polarities [8]. Polymer NPs allow for the encapsulation of molecules that can be released at targeted sites [10,11,12]. Among the inorganic NPs, there are silica NPs (crystalline or amorphous), metal NPs (such as copper, titanium, nickel, selenium, gold, silver), metal oxides (titanium dioxide, iron oxide, zinc oxide, magnesium oxide, etc.) or quantum dots (typically made of semiconducting materials, such as Si). Inorganic NPs have been used to synthesize nanostructured materials for various drug delivery and imaging applications and can have a variety of sizes, structures, and geometries [8,13].

Metal NP nanostructure may be diverse (nanotube, nanorod, nanowire, nanocrystal, spherical, and dendritic aggregated nanomaterial, quantum dots, etc.) among others [8]. Metal NPs can be covered with organic compounds such as polymers to give nanocomposites. The characteristics of nanomaterials (type, shape, size, electrical charge, surface coating, concentration, etc.) are responsible for their effectiveness [14,15]. Characterization of metal NPs is essential to know their mechanism of action and toxicity. For this purpose, different techniques such as UV-visible spectroscopy, X-ray diffraction analysis, scanning electron microscopy (SEM), transmission electron microscopy (TEM), Fourier transform infrared spectroscopy (FTIR), atomic force microscopy, Zeta potential measurement, dynamic light scattering (DLS), or single-particle inductively coupled plasma mass-spectrometry analysis can be used [16,17].

NPs are applied, mainly as drug delivery systems, in different therapeutic areas, such as CNS diseases [18], cardiovascular diseases [19], ocular pathologies [20], Alzheimer’s disease [21], diabetes treatment [22], or immunotherapies [23]. However, oncology is the main area of NP applications [8,24,25], and the second area corresponds to infectious diseases [26,27,28]. An advantage of NP formulations vs conventional systems is their multivalency, such that the presence of various functional groups from a NP permits a higher cell recognition and a higher target binding ability than those of linear polymers [12].

The use of metal NPs to treat infections is particularly interesting against multi-drug resistant (MDR) pathogens [29,30]. Numerous studies have tried to elucidate the mechanisms by which NPs inhibit bacterial growth [29,30,31,32], but a clear and complete understanding has not yet been achieved. The nature of the interaction between different functional groups of bacterial surface and NP surface has been studied [13,33]. Gram-positive and Gram-negative bacterial cell walls have a net negative charge. In Gram-positive, the negative charge is provided by teichoic acids, which are linked to the peptidoglycan or to the underlying plasma membrane. Teichoic acids are anionic owing to the presence of phosphates within their structure. Gram-negative bacteria have in their wall an outer membrane with phospholipids and lipopolysaccharides. Lipopolysaccharides confer a strong negative charge on their surface. Positively charged metal NPs have higher bactericidal activity than negatively charged or neutral metal NPs [34,35]. Thus, many studies have focused on NPs with positive surface charge, particularly AgNPs, which are considered the next generation of antimicrobials for the treatment and prevention of MDR microbes [36,37,38,39]. In addition, they experience slow oxidation and release cations.

Silver nanoparticles (AgNPs) have antibacterial, antiviral, and antifungal properties [25]. The NP nanosize enables interaction with biomolecules on the cell wall and membrane and further infiltration into microbial cells. It is necessary to distinguish between (a) the intrinsic antibacterial properties of NPs, such as the ability to damage bacterial membranes, and cause further damage to molecules or structures (DNA, enzymes, ribosomes, lysosomes, etc.) by diverse mechanisms, or inhibit bacterial biofilm formation, and (b) their properties as drug delivery systems such as the capacity to alter cell wall permeability, permitting the entry of antibiotics, even those attached to the coating shell, inside the cell. In this way, the released drugs can act against the bacteria at the target site of action [40].

**Figure 1:**
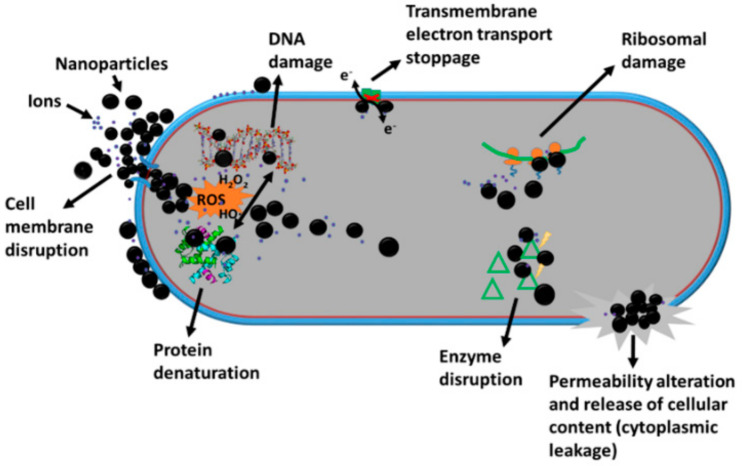
Possible mechanisms of action of nanoparticles in the bacteria.

The mechanisms of action of NPs are not fully understood due to the multi-factorial nature of the activity, making it difficult to decouple the individual mechanisms [30,32]. The main effects of NPs and cations are interactions with the cell membrane of bacteria, which leads to disruption (depolarization of membrane potential), changes its permeability, and allows the intracellular content to be released [30,41]; NPs accumulate in the cell wall, forming “pits” and pores, leading to cell death [32,42]. Released ions inhibit the site between cytochrome α2 and b-cytochromes in the respiratory chain and the cellular respiration process is interrupted in the electron transport chain [30,32,34]. There is inhibition of ribosomal subunits expression, which prevents translation and protein synthesis, inactivation of some cellular proteins and enzymes necessary for adenosine triphosphate (ATP) biosynthesis [30,43] alteration of the normal function of membrane-bound respiratory enzymes, inhibition of thiol group-containing enzymes, such as nicotinamide adenine dinucleotide dehydrogenase II (NADH-dehydrogenase II) in the respiratory system by silver ions [44,45] and generation of reactive oxygen species (ROS), which include hydrogen peroxide (H_2_O_2_), singlet oxygen (O_2_), hydroxyl radical (˙OH), and superoxide radical (O_2_^−^) with oxidative deterioration to cell content [31]. In high levels, these species can damage the DNA [39], and cell membrane, lead to lipid peroxidation and protein oxidation, initiate lethal stress response cascades, and eventually cell death. [13,30,32,39,46] (Figure 1).

There are different approaches regarding the methods of synthesis of metal NPs, overall, there are top-bottom and bottom-up methodologies [25,47]. The former methods start from bulk quantities of materials that are reduced in size and are mixed with clusters of atoms or ions. Some top–bottom methods use physical technologies, such as thermal/laser ablation, mechanical milling, or sputtering. In the bottom-up approach, nanostructures are built atom by atom or particle by particle. This can be attained by a high degree of super saturation followed by nuclei growth [25]. Within the bottom-up approach, there are physical, chemical/electrochemical, and biological methods. Physical methods include condensation, vapor deposition, sol/gel processes, or pyrolysis [47]. In the case of AgNPs, some authors report on chemical synthesis using chemicals, such as NaBH_4_, sodium citrate, chitosan, polyvinylpyrrolidone (PVP), sodium dodecyl sulfate (SDS), etc. that act as reductants and/or capping agents, which are mixed with aqueous solutions of Ag^+^ from soluble silver salts (AgNO_3_) [48,49] under variable conditions of pH/temperature/reaction time to produce NPs. However, most researchers opt for biological synthesis from a variety of natural sources, such as bacteria, fungi, algae, parts of terrestrial plants (leaves, fruits, rhizomes, whole plant, …), sugars, etc. to reduce Ag^+^ and form AgNPs. These biosynthetic methods are considered green or environmentally friendly [25,47,49,50] because hazardous chemicals are not used or residues from the synthetic processes are not released into nature. Moreover, they are not expensive. In particular, the utilization of plant extracts for manufacturing NPs is affordable, readily scaled up, and environment-friendly. Plant extracts have the ability to generate NPs with a specified size, shape, and content. Plant produced NPs have the potential to be extensively employed in current medical processes [47].

The possible potential of the NPs, especially AgNPs, in the control of MDR pathogens has been studied [13,26,32,36,37,40]. However, an accurate assessment of the NP potential in the treatment of infections caused by MDR bacteria, and a critical comparative analysis between reports is not possible, as the methodologies used by researchers are different. No in-depth interlaboratory study using the same methodology, NPs, pathogen, and experimental conditions has been performed, which hinders the correct evaluation of the NP potential to combat these infections. Moreover, despite the advantages and potential applicability of NPs in combating MDR pathogens, their possible toxicity and safety issues have limited their general, efficient, and safe use [51].

Based on the above-cited characteristics of NPs, this review focuses on the current status and future prospects of AgNPs as a possible tool to treat infections caused by some MDR bacteria or bacterial families considered by the WHO [1,2] as priority targets to combat. AgNPs are described as the most efficient antibacterial agents based on their action against the pathogen and their toxicity to the host. The use of nanomaterials as potential antimicrobial agents might be considered a post-antibiotic era, which has the ability to overcome the problem of multi-drug resistance [29]; moreover, to avoid the issue of bacterial resistance to NPs, an understanding of the adaptive mechanisms of microbes to resist the action of NPs should be an objective in future studies [29].

## 2. Survey Methodology

The methodology used to survey the bibliography on the topic reviewed was:

(a) A systematic search on the internet using bibliographic databases (Google Scholar, MedLine, PubMed, Web of Science, Scopus, Springer-link, ScienceDirect, etc.) with the keywords “Nanoparticles”, “nanomaterials”, “Silver nanoparticles” combined with the keywords “multi-drug resistant bacteria”, “*Acinetobacter baumannii*”, “*Pseudomonas aeruginosa*”, “*Staphylococcus aureus*”, “Enterobacteriaceae”, “*E. coli*”, “*Klebsiella*”, “*Salmonella*”, “cytotoxicity”, “toxicity”.

(b) A subsequent search was made using the reference lists on the found articles or reviews. The references were selected on the basis of their relevance to the target microorganisms.

(c) The search had a first introductory part dedicated to performing a general introduction of the usage of nanoparticles in clinical applications, the techniques of characterization of metal NPs, the mechanisms of action, and the methods of synthesis.

(d) The focus was on the utilization of AgNPs to combat the four kinds of microorganisms cited and the toxicological issues (*in vitro* and *in vivo*) related to these NPs.

## 3. *Acinetobacter baumannii*

*Acinetobacter baumannii* is a Gram-negative, aerobic, non-motile bacterium that causes nosocomial infections, most notably ventilator-associated pneumonia, and bacteremia, and less frequently meningitis, skin and soft tissue infections, urinary tract infections, and endocarditis. *A. baumannii* pneumonia and bacteremia are typically acquired in the hospital and mainly affect critically ill patients. These are severe infections for which almost no treatments exist and are associated with high mortality [52,53]. Particularly concerning are pan-drug resistant strains of *A. baumannii*, with resistance to all clinically used antibiotics [4,54,55,56]. Consequently, novel strategies for managing these infections are required. The mechanisms of resistance, virulence, and pathogenicity of MDR *A. baumannii* have been recently reviewed [53].

The use of AgNPs has been explored as one of such strategies. Table 1 lists some of the research activities achieved on the use of AgNPs against *A. baumannii*. Fourteen antibiotics (amikacin, gentamicin, kanamycin, amoxicillin, ampicillin, ceftriaxone, vancomycin, ciprofloxacin, doxycycline, tetracycline, chloramphenicol, trimethoprim, ceftazidime, and penicillin) were mixed with AgNPs (8–12 nm) and assayed against *A. baumannii in vitro*. The bacterium resulted in highly sensitized through a synergistic effect becoming susceptible to antibiotics except for cephalosporins [57]. Biosynthesized AgNPs demonstrated good activity against *A. baumannii* both *in vitro* and *in vivo* by impregnation of cotton fabric and further application to wounds of Sprague–Dawley infected female rats [58]. Biosynthesized spherical AgNPs (27 nm) inhibited the growth of *A. baumannii* more than vancomycin used as a control [59]. AgNPs capped with different compounds have been studied regarding their activity against *A. baumannii* strains. While chitosan and SDS-capped AgNPs were ineffective, citrate and PVP-capped AgNPs showed a good inhibitory effect on such strains. PVP capped AgNPs (PVP-AgNPs) proved very effective against carbapenem-resistant strains of *A. baumannii* [60], being suggested as an alternative to carbapenem, and were successfully assayed in human pulmonary host cells (A-549) at doses that were not toxic to these cells. Thus, they might be used alone or along with carbapenem to cure infections caused by carbapenem-resistant strains of *A. baumannii* [61,62]. A nanocomposite made of AgNPs coated with SH-PEG-NOTA (thiol-containing polyethylene glycol linked to 1,4,7-triazacyclononane-1,4,7-triacetic acid), and imipenem, noted as IPM@AgNPs-PEG-NOTA, was assayed and shown to be a promising antibacterial agent of security, pH sensitivity, and high efficiency in reversing resistance and synergistically combatting carbapenem-resistant *A. baumannii* [63]. Single AgNPs completely inhibited carbapenem-resistant *A. baumannii* growth at 2.5 μg/mL and AgNP treatment showed synergistic effects with polymyxin B and rifampicin, and an additive effect with tigecycline [64]. Minimal inhibitory concentrations (MIC) of AgNPs against various *A. baumannii* strains were found in the 0.39–0.78 µg/mL range, and the more resistant strains were generally less susceptible to antibiotics [65]. AgNPs play an important role in the prevention of burn infections frequently caused by MDR *A. baumannii* because they are more active than AgNO_3_ or sulfadiazine and are less chemically contaminated than ion forms [65]. A xanthan gum polymer containing spherical AgNPs (diameter < 10 nm) was tested *in vitro* on MDR strains of *A. baumannii* which proved sensitive to the Ag nanocomposite [66]. AgNPs show synergism with imipenem and other antibiotics against planktonic cells and biofilms of *A. baumannii* [56,64,65,66,67]. Biofilms are surface-associated bacterial communities that are found embedded in a self-produced exopolysaccharide matrix that attaches to surfaces or living tissues [68]. Trimethyl chitosan-capped AgNPs with positive surface charge inhibited MDR *A. baumannii* strains and other pathogenic bacteria and MIC obtained by the microdilution method were ≤12.25 µg/mL for all the tested strains [48]. An extensive study on the antibacterial activity of AgNPs against *A. baumannii* AIIMS 7 in planktonic and biofilm mode demonstrated that AgNPs inhibited planktonic bacteria at a concentration of 16 μg/mL and exhibited a synergistic interaction with doxycycline, tetracycline, and erythromycin [69] in agreement with other studies [65]. 

The importance of visible light to induce the bactericidal mechanism of AgNPs has been reported by Shi et al. [70], who claimed that this is a key factor to catalyze the massive aggregation of cellular proteins in bacteria without the need for silver ion release or formation of ROS. AgNPs affected bacterial growth, distorted the cellular morphology, and induced intracellular oxidative stress thus rendering these bacteria susceptible to NPs. AgNPs interact with thiol-groups, which indicates their potential to inactivate cellular proteins [71]. AgNPs synthesized and functionalized with two capping agents (3-mercapto-1-propane sulfonate and 1-thio-D-glucose) were active against *A. baumannii* ATCC19606T and other bacteria belonging to the ESKAPE (*Enterococcus faecium, S. aureus, Klebsiella pneumoniae, A. baumannii, P. aeruginosa,* and *Enterobacter* species) group [71]. *In vitro* studies using AgNPs incubated with different antibiotics showed synergistic antibacterial activities against ESKAPE [72]. AgNPs can simultaneously induce apoptosis and inhibit new DNA synthesis in MDR *A. baumannii* in a dose-dependent manner [73]. Hetta et al. [74] assayed the activity of AgNPs against MDR *A. baumannii in vitro*. AgNPs produced marked inhibition zones in all tested bacterial strains (mean = 16 mm and range = 6–27 mm) at a level of 50 μg/mL and even on biofilms; the inhibitory activity was more pronounced on weak biofilm producers and this activity was due to a decrease in the expression of some genes related to the formation of biofilms. Thus, we believe that AgNPs can be useful to combat MDR *A. baumannii*, especially by exploiting their synergy with antibiotics; however, their application in human patients needs more research to resolve the associated toxicological issues, which are reviewed in Section 7.

**Table 1:** Research on the activity of AgNPs against *A. baumannii in vitro*.

Synthetic Method of the AgNPs	AgNP Size (nm)	Particle Shape	Capping	Antibiotic Added	MIC/MBC (µg/mL)	Proposed Mechanism of Action	Ref.
From *Acinetobacter calcoaceticus*	8–12	Spherical	No	AMI, AMP, AMX, CAZ, CHL, CIP CRO, DOX, GEN, KAN, PEN, TET, TMP, VAN	MIC (antibiotics + AgNPs) from <0.015 to 2048, depending on the antibiotic; the lowest MIC for DOX, TET and TMP; CIP (0.125), AMI, GEN, KAN (2); CAZ (512, CRO (2048)	Synergy between AgNPs and antibiotics, except for cephalosporins. No MIC data for CHL and VAN	[57]
From *Cassia fistula* fruit	50–150	Triangular, hexahedral, amorphous	No	No	62 µl/mL (*in vitro* assay)	Mechanism of action was not suggested	[58]
From *Salvia leriifolia* leaf	27 (avg.)	Spherical	No	No	(101.4 ± 2.4)% inhibition *vs* control	Mechanism of action was not suggested	[59]
From PVP	6–10 (TEM)	Spherical	PVP	No. AgNPs were compared with CAR and other antibiotics but not mixed	MIC for IPM: 64 (*vs* a highly resistant strain), 32, 8, 8. Four resistant strains were assayed. No MIC for PVP-AgNPs was provided, but they were active against 3 of the 4 strains	Mechanism of action was not suggested. Reference to previous work	[60]
Reduction with PVP, or Na citrate, or SDS, or chitosan (Chit)	6–10 (PVP); Not indicated (others)	Spherical	PVP, Citrate (CIT), SDS, Chit	AMP, DOR, IPM	64 (highly resistant strain)	Synergy with IPM and DOR, (CIT-AGNPs: IPM (Chit- and SDS-AgNPs). Synergy with DOR and AMP (PVP-AgNPs)	[61]
Reduction with Na citrate; then capping with SH-PEG2000-NOTA	30 (avg.)	Spherical	SH-PEG-NOTA + IPM	IPM	64 (at conc. of 60–100 µg/mL)	Mechanism of action not suggested. Sinergy between AgNPs and IPM	[62]
Reduction with NaBH_4_ + Na citrate	5–12 8.4 (avg.)	Spherical mainly	Citrate	PMB, RIF, TGC	MIC: 2.5 (AgNPs alone), FIC index: 0.19 (PMB), 0.38 (RIF), 0.75 (TGC)	Sinergy with PMB and RIF; additive effect with TIG	[63]
Axonnite^®^ prepared by micro-explosion	2–5 (70–75%); 5–100 (30–25%)	Not indicated	No	No	0.39–0.78	Mechanism of action not indicated	[64]
From *Xanthomonas* spp.	<10	Spherical	Xanthan gum	No	Not indicated	Mechanism of action not indicated	[65]
From *Dioscorea bulbifera*	8–20	Mostly spherical some nanorod, triangle	No	Aminoglycosides, β-lactams, cephalosporins, CAR, PMB, VAN, and others	No MIC was given. Only inhibition diameters on solid phase cultures were provided	Sinergy with β-lactams (mainly PIP) and ERY	[66]
From bacteria	8-12	Variable	NA	DOX, ERY, TET	MIC: 16 (against planktonic cells); MBEC: 2000 (against biofilms)	Synergy with DOX, TET and ERY. Intracellular oxidative stress; interaction with thiol-groups	[69]
Commercial	11.12 ± 0.07	Spherical	PVP	No	MIC: 0.9 (MDRAB) MIC: 2.1 (against a sensitive strain of *A. baumannii* ATCC 19606)	Photocatalytic induction of massive aggregation of cellular proteins under visible light. This process is not dependent on the bacterial species	[70]
Reduction with NaBH_4_. Then mix with 3MPS and TG (variable ratios) or only with 3MPS	3 ± 1, 6 ± 2 or 10 ± 2 (by DLS)/ 15–20 (by TEM)	Spherical	3MPS-TG (two patterns) and 3MPS	No	IC_90_ > 128 for *A. baumannii* ATCC19606	Not reported	[71]
Commercial	5–10	Not indicated	No	15 antibiotics were used for assessing bacterial resistance. They were not mixed with AgNPs.	CFU results showed that 38 MDRAB clinical isolates from hospital patients were sensitive to the AgNPs. MIC and MBC were not given	AgNPs induced apoptosis in MDRAB clinical isolates. This activity increases with increasing AgNP conc. Bacterial DNA synthesis decreases with increasing AgNP level	[73]
Reduction with PVP	10–50	Spherical	Not indicated	No	MIC: 4–25 depending on the ability to produce biofilms more or less strong	AgNPs significantly interrupted bacterial growth and multiplication	[74]

Abbreviations: AMI: amikacin; AMO: amoxicillin; AMP: ampicillin; avg.: average; CAR: carbapenems; CAZ: ceftazidime; CFU: colony forming units; CHL: cholesterol; CIP: ciprofloxacin; CRO: ceftriaxone; DLS: dynamic light scattering; DOR: doripenem; DOX: doxycycline; ERY: erythromycin; FIX: fractional inhibitory concentration; GEN: gentamicin; IC_90_: 90% inhibitory concentration; IPM: imipenem; KAN: kanamycin; MBC: minimum bactericidal concentration; MBEC: minimum biofilm eradication concentration; MDRAB: multiple drug-resistant *Acinetobacter baumannii*; MIC: minimum inhibitory concentration; 3MPS: 3-mercapto-1-propanesulfonate; NA: Not available; NOTA: 1,4,7-triazacyclononane-1,4,7-triacetic acid; PEG: polyethylene glycol; PEN: penicillin; PIP: piperacillin; PMB: polymyxin B; PVP: polyvinylpyrrolidone; RIF: rifampicin; ROS: reactive oxygen species; SDS: sodium dodecyl sulfate; TEM: transmission electron microscopy; TET: tetracycline; TG: 1-thioglucose; TGC: tigecycline; TMP: trimethoprim; VAN: vancomycin.

## 4. *Pseudomonas aeruginosa*

*Pseudomonas aeruginosa* is a Gram-negative bacterium highly susceptible to genetic changes leading to resistance to antimicrobials and the consequent complications in impaired or immunocompromised patients. It can form biofilms [68]. Due to its ability to survive in harsh environments, *P. aeruginosa* is one of the most important agents in nosocomial infections [75] and its disease spectrum continues to expand from urinary tract infection to septicemia, osteomyelitis, and endocarditis, posing new challenges because resistance to current therapy limits the available treatment options [76]. Among the different nanosized antibacterial agents, silver is the most effective because of its broad-spectrum activity against bacteria, viruses, and eukaryotic microorganisms [77]. A summary of the research performed on the usage of AgNPs against *P. aeruginosa* is shown in Table 2. Morones et al. [77] proposed that AgNPs act in three ways against Gram-negative bacteria: (1) AgNPs mainly in the range of 1–10 nm attach to the surface of the cell membrane and drastically disturb its proper functioning, such as permeability and respiration; (2) they can penetrate inside the bacteria and cause further damage by possibly interacting with sulfur- and phosphorus-containing compounds, such as DNA; and (3) AgNPs release Ag^+^ ions, which additionally contribute to the bactericidal effect of the AgNPs. Biogenic AgNPs (60–80 nm size range) showed activity against an antibiotic-resistant strain of *P. aeruginosa* and enhanced the antimicrobial activity of ampicillin, gentamicin, vancomycin, and streptomycin when combined with them [78]. Biosynthesized AgNPs in the 20–50 nm size range also exhibited high antimicrobial activity against this bacterium [79,80]. The MIC and the minimal bactericidal concentration (MBC) for *P. aeruginosa* (6.4 pM) were higher than for other bacterial strains, which could be due to their biofilm-forming ability [80]. The MBC of AgNPs synthesized from *P. putida* (15–40 nm) against *P. aeruginosa* was 1 µg/mL [81]. Spherical silver nanocomposites (5–50 nm) biosynthesized from *Lactococcus lactis* exerted antimicrobial activities against *P. aeruginosa* and the MIC_90_ against this bacterium was 6.25 μg/mL [82]. AgNPs showed synergistic activity against MDR *P. aeruginosa* when combined with antibiotics. AgNP functionalization with ampicillin (AMP-AgNP) showed advantage over non-functionalized AgNPs as they killed ampicillin-resistant strains of *P. aeruginosa* (MBC = 1 µg/mL) [83].

AgNPs can inhibit biofilm formation by *P. aeruginosa* in ocular-related infectious disease microbial keratitis by more than 95% by arresting the synthesis of the exopolysaccharide matrix [84]. Such effectiveness in inhibiting biofilm formation was found at bacterial levels of 10^4^–10^5^ CFU/mL with inhibition rates of 56–67% [85]. The activity of 10-nm size commercial AgNPs against *P. aeruginosa* strains with resistance to some antibiotics was evaluated using concentrations of 0.156–5.0 µg/mL. After 12 h, a dose of 5.0 µg/mL proved very effective (approximately 99.9% bacterial death), even when tested against hospital MDR strains [75]. Habash et al. [86] assessed the efficacy of citrate-capped AgNPs alone and combined with the antibiotic aztreonam against *P. aeruginosa* biofilms *in vitro*. The effects of aztreonam alone were limited or even enhanced biofilm biomass since doses as high as 512 µg/mL had no lasting effect on cell viability within the biofilm. AgNPs (10–20 nm) evaluated individually showed more efficacy than aztreonam in preventing biofilm and planktonic cell recovery. The 10 nm AgNPs alone presented only minor, but significant defects in biofilm architecture, even when alterations in cellular morphology or ultrastructure were not evident. In contrast, AgNPs (40–60 nm) demonstrated limited inhibition of biofilm biomass and viability, while the 100 nm AgNPs showed no major inhibitory effects [86]. Citrate-capped AgNPs proved more effective against *P. aeruginosa* biofilms than applying ionic Ag, which indicates that AgNPs can release small amounts of Ag^+^, thus resulting in higher activity. AgNPs of 8 nm were more efficient in detaching *P. aeruginosa* biofilms than particles of 20 and 35 nm [87]. The biofilm removal effect of AgNPs was size-dependent, as the smaller nanoparticles showed higher effectiveness. Citrate-capped AgNPs (10–20 nm) synergistically potentiated tobramycin activity to inhibit these biofilms, which may be due to the disruption of cellular membranes [88]. Minimum biofilm eradication concentration assays using clinical *P. aeruginosa* strains showed that small AgNPs inhibited biofilms better than larger AgNPs, although the synergy effect is likely strain-dependent. The MIC of AgNPs against this bacterium is in the order of 1–2 μg/mL [46,89]. Thus, the AgNP antibacterial effects against this bacterium are dose- and time-dependent [90,91]. The antibacterial activity of AgNPs is due to the generation of ROS, malondialdehyde, and leakage of proteins and sugars in bacterial cells. Moreover, AgNP-treated bacteria had significantly lower lactate dehydrogenase (LDH) activity and lower adenosine triphosphate levels than the control. Furthermore, AgNP-treated bacteria showed downregulated expression of glutathione, upregulation of glutathione S-transferase, and downregulation of both superoxide dismutase and catalase. These physiological and biochemical measurements observed in AgNP-treated bacteria suggest that AgNPs can induce bacterial cell death. The antibacterial activity of the biosynthesized AgNPs was evaluated against Gram-negative bacteria, such as *E. coli* and *P. aeruginosa* [46] and it was found that AgNPs showed bactericidal rather than bacteriostatic effect; a bactericidal agent is preferred clinically because bacterial killing should produce a faster resolution of the infection, improves clinical outcome, and reduces the likelihood of the emergence of resistance and the spread of infection [90]. Biosynthesized AgNPs (7.1 nm) exhibited antibacterial activity and killed all the cells of this bacterium when treated with 2.7 μg/mL for 4 h [92]. The intracellular ROS production suppressed the antioxidant defense and exerted mechanical damage to the membrane. AgNPs inhibit the catalase and peroxidase activity so that the excessive ROS is not eliminated, which may result in impaired DNA and ribosome and declined synthesis of the macromolecules [91,93]. AgNPs also induce surface charge neutralization and alter the cell membrane permeability, causing non-viability of the cells. The synergistic effect of AgNPs combined with antibiotics against both susceptible and resistant *P. aeruginosa* was evaluated and was shown only against susceptible *P. aeruginosa* [94]. This synergistic effect has been reported not only for *P. aeruginosa* but also for other bacteria [95,96]. However, strains of *P. aeruginosa* resistant to streptomycin and rifampicin retained their resistance when these antibiotics were combined with AgNPs [94]. The activity of biogenic AgNPs alone and combined with antibiotics was evaluated against *P. aeruginosa,* and the lowest MIC found was 16 μg AgNPs/mL [97], which is higher than other reported MIC values [46,95] although differences may be due to differences in size. Electrochemically synthesized AgNPs were able to reduce the biofilm’s viability of *P. aeruginosa* achieving biofilm suppression at a level of 17 μg/mL (4 × MIC) [98]. By using proteomic analysis, it was demonstrated that the mechanisms of the AgNP antibiofilm activity involve interferences with multiple processes of the *P. aeruginosa* biofilm formation, such as bacterial motility, oxidative stress response, iron homeostasis, respiration, and quorum sensing systems [93]. Therefore, AgNPs exhibit a remarkable antibacterial activity against MDR *P. aeruginosa*, representing a possible alternative for antibiotics and they can also be promising antibiofilm agents. The systemic administration of these particles seems at this time difficult to implement owing to the possible accumulation and damage of tissues and organs (toxicity); however, either coating of prosthetic devices/catheters or their topical application for the treatment of skin infections and the prevention of disease in burnt patients may be a future application of AgNPs [99,100]. Therefore, the utility of AgNPs against this bacterium has been evidenced. As in the case of *A. baumannii*, their usefulness in treating human infections requires more research.

## 5. Enterobacteriaceae

The Enterobacteriaceae family is ubiquitous and its members are found worldwide in different ecological sources. Some species are part of the normal flora of animals, including humans, although many are frequently associated with diarrheal disease and extraintestinal infections. This family includes more than 210 species and 53 genera, and these numbers continue to increase. Some of the most important pathogens in human history, such as *Yersinia pestis*, belong to this family. Other pathogens of huge public health concern are *Salmonella enterica* serotype or serovar Typhi, *Shigella* spp., *Klebsiella pneumoniae* and *E. coli.* Other Enterobacteriaceae causing infections in humans include *Citrobacter* spp., *Enterobacter* spp., *Serratia marcescens*, or *Proteus* spp.

A recent problem in the medical field is the increasing number of bacterial strains within the Enterobacteriaceae family able to produce extended-spectrum β-lactamases (ESBL) [101]. The carbapenems are the mainstay of therapy for treating serious and life-threatening infections caused by Enterobacteriaceae producing ESBL, but the emergence of resistance to carbapenems has led to limited therapeutic options. Mechanisms of resistance to carbapenems include the production of β-lactamases, efflux pumps, and mutations that alter the expression and/or function of porins [102].

Using *E. coli* as a model for Gram-negative bacteria, it was proved that negatively charged AgNPs may be used as antimicrobial agents. Table 3 lists some relevant results attained in the research on the application of AgNPs against enterobacteria. Aggregates composed of AgNPs and dead bacterial cells were observed by SEM. The AgNPs interact with elements of the bacterial membrane and damage the cell. TEM analysis and Energy Dispersive Analysis X-ray confirmed the incorporation of AgNPs into the membrane and the formation of pits on the cell surface [103].

The antibacterial efficiency of AgNPs was tested against *E. coli* in solution and solid medium. The AgNPs exhibited antibacterial activity at low levels and they were cytotoxic at a concentration of 8 μg/cm^2^ of solid culture medium [104]. The mechanism behind the antibacterial activity of AgNPs was related to their high surface area/volume ratio. The effectiveness of penicillin G, amoxicillin, erythromycin, clindamycin, and vancomycin against *E. coli* increased in the presence of AgNPs [105]. Highly monodispersed AgNPs (average size 13.5 nm) inhibited *E. coli* growth, with MIC > 3.3 nM, and the inhibition effect was dose-dependent [106]. Biosynthesized AgNPs having an average size of 30.5 nm in a protein matrix were very effective to control the growth of *K. pneumoniae* [107]. Spherical AgNPs (5–40 nm) biosynthesized from *Fusarium acuminatum* showed an efficient antibacterial effect against *E. coli, S.* Typhi, and *Staphylococcus epidermidis*, which was 1.4–1.9 fold stronger than that of Ag^+^ [108]. AgNPs biosynthesized from *E. coli* proved very efficient against *E. coli* giving an MBC of 8 µg/mL [81]. 

Chitosan-capped silver nanocomposites had activity against *E. coli* higher than that of chitosan alone [109,110]. Large AgNPs (160–180 nm diameter) showed only moderate activity against *S.* Typhi and *K. pneumonia* and were inactive against *Vibrio cholera* [111]. Clinical strains of *E. coli*, *K. pneumoniae*, *Enterobacter* sp., and *Proteus morganii* isolated from patients affected by urinary tract infections were treated *in vitro* with AgNPs. *Enterobacter* sp. proved very susceptible but the *E. coli* strain was not affected. This was attributed to the MDR nature of *E. coli* developed by point mutations [112]. However, there is evidence that small AgNPs (5 nm) can inhibit growth and even kill the cells of *E. coli* by destroying the bacterial membranous structure and altering its permeability [113], which agrees with Devina Merin et al. [114] who found that AgNPs provided good results in terms of inhibition of strains of *Klebsiella* sp., *Proteus vulgaris,* and *E. coli*. *Salmonella* Typhimurium was less sensitive to AgNPs (20–30 nm size range) than *E. coli* [115]. Concerning capping agents, PVP-AgNPs showed better antibacterial properties both *in vitro* and *in vivo* than citrate-capped AgNPs [116]. This may be due to the better stability and higher uptake by the cells of the PVP-AgNPs. Cell uptake of capped AgNPs was significantly higher than for uncapped AgNPs in the presence of serum. The antibacterial activity of N-stearoyl ethanolamine (NSEA) capped AgNPs was tested against pathogenic bacteria, such as *E. coli*, *S.* Typhi, *Shigella* spp., and *K. pneumoniae*, and their estimated MIC values ranged from 6 to 12 μg/mL [117]. The formation of ROS in *E. coli* after damage to the cell surface has been suggested as a cause of bacterial toxicity due to the activity of AgNPs [118].

As indicated in previous sections, synergistic effects between AgNPs and some antibiotics also occur with enterobacteria [78,96,119,120,121]. AgNPs showed antibacterial activity against MDR strains that produce a broad spectrum of β-lactamases or carbapenemase (ESBL-positive *E. coli*, ESBL-positive *K. pneumoniae*, AmpC-positive *E. coli,* and *K. pneumoniae*-carbapenemase (KPC)-positive *K. pneumoniae*) when combined with cefotaxime, ceftazidime, meropenem, ciprofloxacin, and gentamicin, as reflected by low MIC values [96]. The strongest activity was demonstrated against ESBL-positive *E. coli*, as only 0.8 mg AgNPs/L killed the bacteria while the highest MIC (6.75 mg/L) was required to kill ESBL-positive *K. pneumoniae*. This study of the antibacterial activity of AgNPs combined with antibiotics confirmed the existence of a synergistic effect resulting from the combination of these two antimicrobial agents. A lack of β-lactamase production in bacteria when antibiotics were combined with AgNPs confirmed the restoration of the antibacterial effect of antibiotics in the presence of Ag [96].

As found for *P. aeruginosa*, AMP-AgNPs have also an advantage over enterobacteria because they kill ampicillin-resistant strains of *E. coli*. MBCs were 1 µg/mL against *E. coli*, 2 µg/mL against *V. cholera,* and 4 µg/mL against *E. aerogenes* [83]. Regarding the mechanism of action of AgNPs against *E. coli*, the stabilities of the antibacterial activity under various pH-values and temperature conditions, the protein leakage caused by increased membrane permeability, and the inactivation of LDH due to the nanoparticle-induced formation of ROS were demonstrated [122]. The antibacterial activities of ampicillin, kanamycin, erythromycin, and chloramphenicol increased in the presence of AgNPs against test strains [120]. AgNP-loaded TiO_2_ nanotube arrays were fabricated on titanium implants for a customized release of Ag^+^. The antibacterial properties of silver nanotubular structures combined with vancomycin, rifampicin, gentamicin, and levofloxacin were tested *in vitro*. Improved effectiveness of the combined therapy was observed for all tested bacterial strains, including *E. coli*. After the treatment, experiments further proved the synergistic antibacterial effect both *in vitro* and *in vivo* [121]. Although bacterial resistance to antibiotics is well-known, and bacterial resistance to Ag has been reported [122], the possible development of resistance to AgNPs has not been fully explored. Susceptible strains of *E. coli* and other bacteria were converted into AgNP-resistant strains by culturing them in agar media containing AgNPs until a concentration near or over the MIC was reached on which the bacteria could grow [90]. AgNPs (10–25 nm) mostly spherical were assayed against enterobacteria (*Salmonella* spp. and *Shigella* spp.) isolated from poultry feces; at a concentration of 16 μg/mL AgNPs showed bacteriostatic and bactericidal effects against *S.* Montevideo, *Shigella sonnei*, and *S. enteritidis* but at a concentration of 8 μg/mL the nanoparticles had both bacteriostatic and bactericidal effects in the case of *S.* Poona, *Shigella boydii*, and *S.* Typhimurium [123]. Lower MIC and MBC values (0.085 ± 0.126 μg/mL and 0.508 ± 0.315 μg/mL, respectively) were obtained for 20 MDR *Salmonella* spp. strains recovered from feces of diarrheal sheep and goats when treated with PVP-capped chemically synthesized AgNPs (2.95–12.2 nm size range). Interestingly, an *in vivo* assay on a mouse model showed that AgNPs had no toxic or pathologic effects [124]. In a recent study, biosynthesized AgNPs from *Massilia* sp. showed strong antimicrobial activity against *K. pneumoniae* and *S. aureus enteritidis*. The MICs of biosynthesized AgNPs against *K. pneumoniae* and *S. enteritidis* were 12.5 and 25.0 μg/mL, respectively while the MBC against both pathogens was 50.0 μg/mL [125]. A study on the susceptibility of three different *Salmonella* serovars (Enteritidis, Hadar, and Senftenberg) to AgNPs showed an immediate, time-limited, and serovar-dependent reduction of bacterial viability [126]. For *S.* Senftenberg, the reduction was observed for up to 4 h of incubation in the presence of 200 µg AgNPs/mL; on the contrary, *S.* Enteritidis and *S.* Hadar were inhibited for up to 48 h. Thus, success is strongly *Salmonella* strain-dependent, since great differences in terms of effective dose and time of action were observed for the examined serovars. Reverse transcription and PCR experiments demonstrated the constitutive expression of the plasmidic silver resistance determinant (SilB) by *S.* Senftenberg, thus suggesting the importance of a cautious use of AgNPs [126]. The use of AgNPs conjugated with antimicrobial peptides such as andersonin Y1 (AY1) and two AY1-cysteine derivatives has been recently explored and found useful as a new strategy to combat MDR bacteria (*P. aeruginosa*, *E. coli*, *K. pneumoniae,* and *S.* Typhi) [127].

The mechanism of action of Ag/AgCl NPs is similar for different enterobacteria (*Serratia marcescens*, a strain of *K. pneumoniae* carbapenemase-producer, a strain of ESBL *K. pneumoniae* and *E. coli* ATCC 25922) and independent of the presence of enzymatic mechanisms of resistance to β-lactamase. This finding was confirmed by the MIC determination since all bacterial strains showed the same sensitivity profile (MIC = 10.52 μg of Ag/AgCl NPs/mL) [128]. Two *E. coli* strains developed resistance to AgNPs after repeated exposure due to the production of the adhesive flagellum protein flagellin, which triggers nanoparticle aggregation. Resistance evolved without any genetic changes; only phenotypic change was needed to reduce the NP colloidal stability and eliminate their antibacterial activity. The resistance mechanism was not overcome by additional stabilization of AgNPs using surfactants or polymers but it was strongly suppressed by inhibiting flagellin production with pomegranate rind extract [129]. The antibacterial usefulness of AgNPs against some of the most important species of the Enterobacteriaceae family has been put in evidence but its clinical application requires further research as previously indicated for other bacteria.

**Table 2:** Research on the activity of AgNPs against *Pseudomonas aeruginosa in vitro*.

Synthetic Method of the AgNPs	AgNP Size (nm)	Particle Shape	Capping	Antibiotic Added	MIC/MBC (µg/mL)	Proposed Mechanism of Action	Ref.
Commercial in a carbon matrix	16 ± 8	Cub-octahedral, multiple-twinned icosahedral and decahedral	No	No	Not provided	Disruption of bacterial membrane altering permeability/respiration; damage of S and P containing compounds (DNA); AgNPs release Ag^+^	[77]
From *Phoma glomerata*	60–80	Spherical	Bio-molecules	AMP, GEN, KAN, STR, VAN	Not provided	Synergy with AMP, GEN, VAN, and STR	[78]
From *Pseudomonas aeruginosa*	20–50	Spherical	Not indicated	No	50	Reference to the mechanisms of action given in ref. [77]	[79]
From *Pseudomonas aeruginosa*	25–45	Spherical	Not indicated	No	MEC: 6.4 pM MBC: 6.4 pM	Reference to previously reported mechanisms. Effect on the membrane: release of Ag^+^	[80]
From *Pseudomonas putida*	15–40	Spherical, truncated triangle, triangle, and hexagonal	Biological corona	No	MIC: 1	Reference to the same mechanisms given in ref. [77]	[81]
From *Lactococcus lactis* 56	5–50; avg. 19 ± 2	Spherical	Organic material from *L. lactis*	No	6.25	No mechanism of action was proposed	[82]
Reduction with NaBH_4_ + Na citrate	4	Not indicated	Citrate/AMP	AMP linked to AgNP, (AMP-AgNP)	MBC: 1 for AMP-AgNPs, against all the tested bacteria, four times lower than AgNPs alone (MBC: 4)	No mechanism of action was proposed	[83]
From *Bacillus licheniformis*	50 (avg.)	Spherical	Not indicated	No	A concentration of 100 nM inhibits 95–98% biofilm	Biofilm inhibition by arresting the synthesis of the exopolysaccharide matrix	[84]
Commercial	20–30	Not indicated	Not indicated	No	20 µg/mL inhibits about 56% biofilm of MDR *P. aeruginosa* and 67% of other strain	Biofilm inhibition. Mechanism of action was not provided	[85]
Commercial	10, 20, 40, 60, and 100	Not provided	Citrate	ATM	MIC against planktonic cells of AgNPs alone: from 0.234 (10 nm particles) to 7.50 (100 nm particles)	Synergistic effects of AgNP/ATM against biofilms are size-dependent. Optimal size: 10 nm, followed by 20 nm; worst size: 100 nm	[86]
Commercial	10	Not provided	Citrate	No	5, approx. 99.9% MDR *P. aeruginosa* death	Suggested mechanisms of action are those proposed in refs. [77,103,113]	[75]
(1) Reduction with gallic acid; (2) growth of small AgNP nuclei by citrate/ascorbic acid addition at various Ag^+^/Ag^0^ ratios	8 (avg.) before seeding; then up to 66 nm. Sizes used in experiments were 8, 20 and 35 nm	Spherical; pseudo-spherical, cylindrical.Other shapes after growth	Citrate	No	600 µg AgNPs/mL produced biofilm detachment in 90% (8-nm AgNPs), and lower % (20- and 35-nm AgNps) depending on the media used	Effectiveness is size related. Low sizes are more effective than high sizes against biofilms. The low efficacy of AgNPs in this study may be due to citrate capping. AgNPs are more efficient than silver ions. Attachment of the NPs onto the microbial cell membrane leads to increased permeability, inhibition of cell wall synthesis, plasmolysis, and cell death	[87]
Commercial	10, 20, 40, 60, and 100	Not provided	Citrate	TOB	MIC against planktonic bacteria: 0.156–0.625 (10 nm); 0.312–2.5 (20 nm); 2.50–10 (100 nm). MEBC against biofilms: 1.25–5.0 (10 and 20 nm); 2.5–>10 (40 nm); 5.0–>10 (60 nm)	Synergistic effect (10, 20, 40, 60 nm). Additive effect: 100 nm. The efficacy to inhibit biofilms and planktonic cells is dependent on strain and it is higher for smaller AgNPs either alone or combined with TOB	[88]
Quercetin	11	Spherical	Quercetin	No	MIC: 1	Antibacterial activity due to membrane disruption, generation of malondialdehyde and ROS, and leakage of proteins and sugars in cells. Found in treated cells: downregulated expression of glutathione, upregulation of glutathione S-transferase, downregulation of superoxide dismutase and catalase; inactivation of respiratory chain; low lactate dehydrogenase activity, and low adenosine triphosphate	[46]
From *A. baumannii*	37–168	Spherical	Not indicated	No	MIC: 1.56	Not provided	[89]
Commercial	100	Not indicated	No	No	MIC: 83.3 (±16.7) mM MBC: 83.3–100 mM Anti-biofilm activity not reported	It is suggested a mode of action of AgNPs previously reported and similar to that of Ag^+^, which complex groups containing S, O, or N atoms that are present as thiols or phosphates on amino acids and nucleic acids, ROS production, membrane destabilization, etc.	[90]
Commercial from cyclodextrin	5–20, mostly 5–10 (chosen for the experiment)	Near spherical	No indicated	No	MIC: 1.406–5.625; MBC: 2.813–5.625	The cell wall becomes thin; the cell membrane shrivels and fractures. Production of excessive ROS (oxidative stress); destruction of the redox homeostasis; alteration of the activity of the redox relevant enzymes; apoptosis-like effect. Activity is dose- and time-dependent	[91]
From protein cell-free extract of *Rhizopus oryzae*	9.2 (avg.)	Spherical	Protein corona	No	MIC: 2.25 ± 0.2 MBC: 2.7 ± 0.2	Excessive ROS production. Cell membrane permeability is affected. Membrane destabilization by ROS can be responsible for surface charge neutralization leading to cellular material leakage and cell death. Damages due to AgNP interaction with intracellular proteins and nucleic acids	[92]
Commercial from cyclodextrin	5–20, mostly 5–10 (chosen for the experiment)	Near spherical	No indicated	No	6.25 prevents biofilm formation	Biofilm damage is dose-dependent. AgNPs may induce downregulation of flagellins, fimbrillins, and other proteins of biofilms. Bacterial adhesion and motility are inhibited. The iron homeostasis is disturbed. Excessive ROS can cause lipid peroxidation, impairment of DNA and ribosomes, reduction in synthesis of macromolecules, and bacterial death. Respiratory enzymes are affected, which conducts to hypoxia. ROS production may influence the QS system and inhibit the expression of the virulent factors	[93]
High-voltage method	2–35	Not indicated	No	AMP, CIP, CTZ, MEM, OXA, RIF, STR, TET	MIC: 1; MBIC: 4 (for AgNPs alone)	Synergistic interaction with AMP, STR, RIF, and TET. No interaction with the remaining antibiotics (planktonic cells). The synergistic interactions depend on the doses. No interaction concerning biofilm formation was observed. AgNPs induced synthesis of bacterial DnaK chaperone, but HtpG chaperone synthesis was unaffected	[94]
Reduction of [Ag(NH_3_)_2_]^+^ by D-maltose (modified Tollens process)	26	Not indicated	No	AMI, ATM, CFP, CIP, CST, CTZ, FEP, GEN, MEM, OFX, PIP, TZP	MIC: 7.5 (AgNPs alone)	Synergistic effect of antibiotics combined with AgNPs	[95]
From *Streptomyces xinghaiensis*	5–20 (TEM) 64 (avg.) (nano tracking analysis)	Spherical	Not provided	AMP, KAN, TET	MIC: 16 and MBC: 32 (AgNPs alone)	No interaction between AgNPs and the tested antibiotics is reported	[97]
Electrochemical process	55.6 ± 2.9	Quasi-spherical	No	TOB	MIC: 1.07–4.25 and MBC: 2.125–4.25 (for AgNPs alone)	AgNPs exhibited a comparable or higher antibacterial activity compared to TOB including anti-biofilm activity. AgNPs showed a dose-dependent effect and caused biofilm eradication at a concentration of 4 × MIC. They deconstructed the exopolysaccharide matrix and produced cell lysis	[98]
From (a) leaf extract of *Citrus latifolia;* or (b) from *Aspergillus flavus*	5–70, mostly in the range 20–30	Spherical	No	AMI, CAZ, CIP, KAN, LVX, MEM, TZP	AgNPs alone, MIC: 4–128; for AgNPs from *A. flavus*: or 8–>128 for AgNPs from *C. latifolia*	Damage to the cell wall, membrane, and DNA, induction of ROS production. AgNPs derived from *A. flavus* showed synergistic effects with MEM and LVX	[99]

Abbreviations: AMI: amikacin; AMP: ampicillin; ATM: aztreonam; avg.: average; CAZ: ceftazidime; CFP: cefoperazone; CIP: ciprofloxacin; CST: colistin; FEP: cefepime; GEN: gentamicin; KAN: kanamycin; LVX: levofloxacin; MBC: minimum bactericidal concentration; MBIC: minimum biofilm inhibitory concentration; MEBC: minimum eradication biofilm concentration; MDR: multi-drug resistant; MEM: meropenem; MIC: minimum inhibitory concentration; mM: millimolar; OFX: ofloxacin; OXA: oxacillin; PIP: piperacillin; pM: picomolar; QS: quorum sensing; RIF: rifampicin; ROS: reactive oxygen species; STR: streptomycin; TEM: transmission electron microscopy; TET: tetracycline; TOB: tobramycin; TZP: piperacillin/tazobactam; VAN: vancomycin.

**Table 3:** Research on the activity of AgNPs against Enterobacteriaceae *in vitro*.

Synthetic Method of the AgNPs	AgNP Size (nm)	Particle Shape	Capping	Antibiotic Added	MIC/MEB (µg/mL)	Proposed Mechanism of Action	Ref.
Ascorbic acid + Daxad^®^ 19	12 (mode)	Not indicated	Not indicated	No	MIC: 50–60 (against *E. coli*)	Formation of ‘pits’ in the bacterial cell wall. AgNPs accumulate on the cell wall/membrane and in the cells. Leaking of intracellular substances	[103]
From *K. pneumoniae*	5–32, 22.5 (avg.)	Not indicated	Not indicated	Yes	Not determined. Assays on solid media. Inhibition diameters measured	No mechanism of action was proposed. AgNPs + antibiotics increase the inhibition zone more than some antibiotics alone	[105]
Reduction with NaBH_4_	4–20, 13.4 (avg.)	Not indicated	Not indicated	No	MIC: 3.3–6.6 nM (against *E. coli*)	Formation of free radicals on the AgNP surface and free radical-induced membrane damage	[106]
From spent mushroom substrate	30.5 ± 4.0	Spherical	Proteins	No	Not determined	No mechanism was proposed. Antibacterial properties against *K. pneumoniae* increased with AgNP concentration	[107]
From *Fusarium acuminatum*	5–40; 13 (avg.)	Spherical	Not indicated	No	Not determined. *E. coli* and *S.* Typhi were inhibited, but the efficacy was low	Reference to mechanisms proposed in ref. [103]. The reaction of silver with SH groups of proteins in the cell inactivates proteins	[108]
From chitosan solution + NaOH producing chitosan-AgNP composites	2–4 (90%)	Spherical	Not indicated	No	AgNP-chitosan composite: MIC: 100; MBC: 120 (against *E. coli*)	Destabilization of the bacterial cell wall by the composite. The binding of AgNPs to thiol-containing proteins present in the cell wall leads to penetration. The composite was more efficient than AgNPs or chitosan alone for inactivating bacteria, possibly due to a synergistic effect	[109]
From chitosan solution	4–18, 6–8 (50%)	Not indicated	Not indicated	No	AgNP-chitosan; MIC: 10; MEB: 10 (against *E. coli*)	Chitosan-based AgNPs have a dual mechanism of action for antibacterial activity, the bactericidal effect of AgNPs, and the cationic effects of chitosan	[110]
From *S. aureus*	160–180	Not indicated	Not indicated	No	MIC/MBC were not given. *S.* Typhi and *K. pneumoniae* showed low susceptibility; *V. cholera* was not susceptible	No mechanism of action was proposed	[111]
Commercial	5	Not indicated	Not indicated	No	MIC: 10 (against *E. coli*)	AgNPs accelerate the reducing sugars/protein leakage from the cytoplasm in *E. coli*. The activity of respiratory chain dehydrogenases decreases with time. Cell membranes were severely damaged	[113]
From various marine microalgae	Not indicated	Not indicated	Probably proteins from the source	No	MIC was not given. Inhibition against *Klebsiella* spp., *Proteus vulgaris* and *E. coli.*	No mechanism of action was proposed	[114]
From *Streptomyces hygroscopicus*	20–30	Spherical	Not indicated	No	MIC/MBC not given. The highest antimicrobial activity was against *E. coli.* Lower activity was found against *S.* Typhimurium	No mechanism of action was proposed	[115]
Reduction with: (a) NaBH_4,_ (b) Na citrate_,_ (c) Ethylene glycol + PVP	(a) 75 ± 4.5 (b) 82 ± 5.2 (c) 86 ± 6.7	Not indicated	(a) Uncapped (b) Citrate (CIT) (c) PVP	No	MIC: 6–6.33 (against *S.* Typhimurium); 6.33–6.83 (against *S.* Typhi); 6.67–7 (against *Shigella flexneri*).	No mechanism was proposed. The antibacterial activity was in the order PVP-AgNPs > CIT-AgNPs >> uncapped AgNPs	[116]
NaBH_4_ + N-acylethanolamine	Not indicated	Spherical	N-acylethanol-amine	No	MIC: 6.67 (against *K. pneumoniae*); 7.22 (against *Shigella* sp.); 7.22 (against S. Typhi); 9.06 (against *E. coli*)	No antibacterial mechanism was proposed	[117]
From dried powder of *Ocimum gratissimum* leaf extract	16 ± 2 (TEM)	Triangular	Proteins from the source	No	MIC: 4; MBC: 8 (against MDR *E. coli*)	Intracellular ROS generation; membrane was fragmentary. Inhibition of biofilm formation	[118]
From *Phoma glomerata*	60–80	Spherical	Biomolecules	AMP, GEN, KAN, STR, VAN	Not provided	AgNPs enhanced the antimicrobial activity of antibiotics against *E. coli*. Synergy with AMP, GEN, KAN, VAN, and STR	[78]
Reduction of [Ag(NH_3_)_2_]^+^ by D-maltose (modified Tollens process)	26	Not indicated	No	CIP, CTX, CTZ, GEN MER	MIC AgNPs alone: 0.8 (ESBL-positive *E. coli*); 3.4 (AmpC-positive *E. coli* and KPC-positive *K. pneumoniae*); 6.8 (ESBL-positive *K. pneumoniae*)	Synergistic effects of antibiotics combined with AgNPs (< 1 µg/mL) against multi-resistant enterobacteria that produce broad-spectrum -lactamases or carbapenemase	[96]
Ascorbic acid + Daxad^®^ 19	20	Cubic	AMX linked to AgNPs (supposedly)	AMX	MIC AgNPs alone: 40 (against *E. coli*)	Synergistic effects that may be caused by (a) bonding between AMX and AgNPs or (b) AgNPs can act as carriers of AMX	[119]
From *Trichoderma viride*	20–40	Variable, spherical and other shapes	Not indicated	AMP, CHL, ERY, KAN	MIC (AgNPs alone): 30 (against *E. coli*); 35 (against *S.* Typhi)	Synergistic effects with all the assayed antibiotics. The effectivity order was AMP > KAN > ERY > CHL	[120]
Commercial	Not indicated	Not indicated	No	No	MIC: 100 (against *E. coli*)	Bacterial protein leakage by increasing the membrane permeability. Formation of ROS that inactivate LDH	[122]
Reduction with Na citrate + PVP	10–25	Most spherical, some prismatic	Not indicated (probably citrate/PVP)	No	MIC: 8–16; MBC: 8–16 (against *Salmonella* spp. and *Shigella* spp.)	No antibacterial mechanism was proposed	[123]
Reduction with: NaBH_4_, addition of Na citrate + PVP	6.8 ± 2.28	Spherical	PVP	No	MIC ≤ 0.002–0.313 (MBC: 0.078–1.250 (against *Salmonella* spp. 7 species, 20 strains)	No antibacterial mechanism was proposed	[124]
From bacteria *Massilia* sp.	15–55, 23.2 (avg.) by TEM; 109.3 (avg.) by DLS	Most spherical	Conjugated molecules not specified	No	MIC: 12.5 (against *K. pneumoniae*) and 25.0 (against *S*. Enteritidis); MBC: 50 (against both bacteria)	Morphological damage and distortion of the cell wall of both species. It can be attributed to oxidative stress due to the formation of ROS causing membrane detachment	[125]
Commercial	5 to > 500, mostly 6–20. Two populations within the range	Most spherical, but also polygonal	No	No	Not determined against 3 *Salmonella* serovars: Senftenberg, Hadar, and Enteritidis. Best conc. 200 µg/mL. AgNPs were most effective against *S*. Enteritidis, and not effective against *S.* Senftenberg	The surface area of AgNPs is important for their activity, as Ag^+^ release, the determining factor for antimicrobial activity, might be dependent on the surface area (importance of the AgNP shape)	[126]
Reduction with NaBH_4_ and Na citrate. Further conjugation with peptides	10 (avg.)	Not indicated	Citrate. Then conjugation with peptides AY1 and two AY1 cysteine derivatives at the two terminal positions (C and N) of AY1: AY1C and CAY1	No	MIC_80%_: ~50 µM (AY1-NP), 12 µM (AY1C-NP), 10 µM (CAY1-NP) against *E. coli*; 10 µM (AY1C-NP), 5 µM (CAY1-NP) against *K. pneumoniae*; 15 µM (AY1C-NP and CAY1-NP) against *S.* Typhi	Cell membrane rupture by nano-conjugates. It is suggested that there exists an interaction of peptides with negatively charged phosphate head groups of lipid moieties as well as with water molecules. Interaction with the hydrophobic tails of the membrane produces pores. Then, AgNPs attach the DNA	[127]
From *Fusarium oxysporum.* Ag/AgCl-NP produced	55 ± 18 (TEM); 89 (DLS)	Pseudo-spherical	Proteins	IPM	MIC of Ag/AgCl-NPs: 10.52 for all the bacteria tested (ESBL and *K. pneumoniae* carbapenemase-KPC	Ag/AgCl-NPs + IPM were more active than IPM alone, but no synergistic effect is deduced from the inhibition diameters	[128]
According to refs. [95,96]	26	Not indicated	No	No	MIC increased for *E. coli* CCM 3954 from 3.38 to > 54 after 9 successive cultures and for *E. coli* 013 from 13.5 to > 54 after 14 successive cultures	The increasing MIC values show the gradual development of bacterial resistance against AgNPs, not against Ag^+^. Bacteria repeatedly exposed to sub-inhibitory concentrations of AgNPs can rapidly develop resistance to their antibiotic activity. Resistance is due production of flagellin, a protein of the bacterial flagellum, which causes AgNP aggregation	[129]

Abbreviations: AMX: amoxicillin; avg.: average; AY1: andersonin-Y1; AY1C: andersonin-Y1-cysteine derivative at C-terminus; CTX: cefotaxime; CHL: chloramphenicol; CAY1: andersonin-Y1-cysteine derivative at N-terminus; DLS: dynamic light scattering; ERY: erythromycin; ESBL: Extended-spectrum beta-lactamase; IPM: imipenem; KAN: kanamycin; KPC: *Klebsiella pneumoniae* carbapenemase; LDH: lactate dehydrogenase; MBC: minimum bactericidal concentration; MIC: minimum inhibitory concentration; MIC_80%_: minimal concentration that kills 80% of bacteria; µM: micromolar; nM: nanomolar; PVP: polyvinylpyrrolidone; ROS: reactive oxygen species; TEM: transmission electron microscopy.

## 6. *Staphylococcus aureus*

*Staphylococcus aureus* is a Gram-positive coccus whose cells tend to occur either singly or forming pairs, tetrads, and distinctive irregular “grape-like” structures. Humans are usually colonized by *S. aureus* on external skin surfaces and the upper respiratory tract, particularly the nasal passages. Resistant strains typically produced β-lactamase, which inactivated the β-lactam antibiotics. Efforts were made to synthesize penicillin derivatives that were resistant to β-lactamase hydrolysis. This was achieved in 1959 with the synthesis of methicillin, which had the phenol group of benzylpenicillin disubstituted with methoxy groups [130]. In 1961 there were reports from the United Kingdom of *S. aureus* isolates that had acquired resistance to methicillin (methicillin-resistant *S. aureus*, MRSA), and MRSA isolates were soon recovered from other countries. MRSA is now a problem in hospitals worldwide and is increasingly recovered from nursing homes and the community [131]. *S. aureus* has also become resistant to other last-resort antibiotics, such as vancomycin, daptomycin, and linezolid [132].

Table 4 lists the most relevant results obtained by the application of AgNPs against *S. aureus in vitro*. 

AgNPs can inhibit *S. aureus* growth at concentrations > 33 nM, around ten times higher than those required by *E. coli* [106]. Biosynthesized AgNPs performed successful antimicrobial testing against *S. aureus* [107,133]. AgNPs may be applied to MRSA, the main cause of nosocomial infections worldwide [90,132,134,135,136,137]. AgNPs at sublethal doses together with ampicillin act synergistically against MRSA, with the effect being more pronounced when a lower concentration of ampicillin is present [136]. Synergy effects on MRSA between streptomycin and AgNPs and other nanoparticles have been reported recently [138].

AgNPs act as potential antimicrobial agents and help to inhibit biofilm formation by MRSA and vancomycin-resistant *S. aureus* (VRSA) [139]. According to Ayala-Núñez et al. [134], AgNPs inhibit bacterial growth of both MRSA and non-MR *S. aureus* in a bactericidal rather than a bacteriostatic manner (MBC/MIC ratio  ≤  4). Nanosilver size mediates MRSA inhibition and the cytotoxicity to human cells, being smaller NPs the ones with better antibacterial activity and nontoxic effect on human cells *in vitro*. As in the case of *E. coli,* chitosan-based AgNPs harbored high antibacterial activity against *S. aureus* [110]. The bactericidal effects of AgNPs are not affected by drug-resistant mechanisms of MRSA. AgNPs generate oxidative stress in *S. aureus* mediated by an increase of ROS, which can cause high levels of oxidized proteins and lipids, DNA fragmentation, and modification in membrane potential [122]. It has been found that a strong interaction between AgNPs and the peptidoglycan layer exists and that AgNPs interact with bacterial cell walls individually or via Ag^+^ release generating “pits”. Thereafter, AgNPs accumulate and connect more strongly with underlying layers, also releasing Ag^+^. These phenomena influence the destruction of Gram-positive bacteria more than the damage of Gram-negative bacteria because of the thicker peptidoglycan layer [140].

A MBC of 20 µg/mL was determined for AgNP against *S. aureus*. When *S. aureus* cells were exposed to 50 µg AgNPs/mL for 6 h, the cell DNA was condensed to a tension state and could have lost its replicating abilities and when cells were exposed for 12 h, the cell wall was broken, and the cellular contents were released into the environment. The protein content was highly altered as well [141]. Biosynthesized AgNPs showed enhanced quenching activity against *S. aureus* biofilm and prevented biofilm formation while a synergistic effect of AgNPs with antibiotics (gentamicin, chloramphenicol) in biofilm quenching was effective [142]. The antibacterial activities of penicillin G, amoxicillin, erythromycin, clindamycin, and vancomycin against a test strain of *S. aureus* increased in the presence of AgNPs and the highest enhancing effects were observed for vancomycin, amoxicillin, and penicillin G [105]. The synergistic activity of some of these antibiotics and AgNPs against MRSA was relatively lower than against Gram-negative bacteria [78]. AMP-AgNPs destroy MRSA isolates [83], and to increase knowledge on this topic, AgNPs combined with gentamicin and oxacillin were tested against an MRSA isolate. The activity of these antibiotics increased in the presence of AgNPs, which has been attributed to the interaction of the AgNPs with hydroxyl and amide groups present in the antibiotic molecules [143]. Considering the clinical importance of *S. aureus* and the global emergence of MRSA, the inhibitory effects of AgNPs on growth and capsule formation as a virulence factor of this microorganism were investigated. The AgNP-mediated formation of ROS has been detected in *S. aureus* cells [122]. Consequently, bacterial cell membrane, protein structure, and the intracellular system can be damaged, which could enhance protein leakage by increasing the membrane permeability and decreasing the activity of LDH. The growth and reproduction of AgNP-treated bacteria were quickly inhibited and the pH and temperature conditions did not affect the growth of the treated bacteria [122]. After comparing the activity of AgNPs against *S. aureus* and *E. coli,* the lower efficacy of the AgNPs against *S. aureus* was attributed to differences in the membrane structure. Abbaszadegan et al. [35] found that positively charged NPs were more effective against all tested bacterial species (*S. aureus* and other Gram-positive and Gram-negative bacteria) than neutral or negatively charged NPs. Stable, well-defined AgNPs, mostly spherical in shape (15 nm) were very active against MRSA isolates and coagulase-negative staphylococci in HIV patients while the *in vitro* toxicity was scarce and growth inhibition was dose-dependent as usual [144]. According to current research regarding the synergistic effect of AgNPs and antibiotics, it may be expected that combinations of AgNPs and antibiotics (mainly AMP) including the addition of carrier/polymer for a more effective delivery system to the target site of MRSA, can enhance antimicrobial activity and decrease the toxicity of the separate components [137].

**Table 4:** Research on the activity of AgNPs against *S. aureus in vitro*.

Synthetic Method of the AgNPs	AgNP Size (nm)	Particle Shape	Capping	Antibiotic Added	MIC/MBC (µg/mL)	Proposed Mechanism of Action	Ref.
Microwave AgNO_3_ solution, 1000 W, 15 s	0.5–24, 1 (avg.)	Not indicated	Not indicated	No	MIC: 12.5 (against MSSA and MRSA)	Unequal AgNP distribution on the exterior (9.5–33 nm) and interior (5–9 nm) of the bacteria. Reduction of the PG layer generates destabilization and permeabilization of the bacterial cell membrane and causes osmotic rupture and lysis	[132]
Reduction with NaBH_4_	4–20, 13.4 (avg.)	Not indicated	Not indicated	No	MIC > 33 nM	Formation of free radicals on the AgNP surface and free radical-induced membrane damage	[106]
From plant *Gynura procumbens* aqueous extract. Then, AgNPs were mixed with fungal chitosan (FCS)	10–100	Spherical, triangle, and hexagonal	Chitosan encapsulating AgNP	No	MIC: 4.08 ± 0.47	No mechanism of action was suggested	[133]
Commercial (two sources, a and b)	(a) ~100; (b) 10, 30–40	Not indicated	No	No	10 nm, MIC_99_: 1800; MBC: 2700; 30–40 nm, MIC_99_: 10790; MBC: 10790; ~100 nm, MIC_99_: 2250; MBC: 8990 (against MRSA)	No mechanism of action was suggested	[134]
Reduction with NaBH4, + polyvinyl alcohol (PVA)	9	Not indicated	Not indicated	No	MIC: 1.95; MBC: 3.91 (against MRSA and *S. aureus*)	No mechanism of action was suggested	[135]
From leaves of *Ricinus communis*	7.25	Spherical or oval	Conjugated with STR	STR	MIC of STR-AgNPs: 3.12 ± 0.9 (*S. aureus*)	Synergistic effect of AgNPs and STR	[138]
Reduction with Na citrate, + polyvinyl alcohol (PVA)	17 (avg.)	Spherical	Not indicated	No	MIC: 2; MBC: 4	AgNPs changed the secondary structure (a-helix) of the bacterial cell wall and destroyed its primary structure with the formation of pits, the release of Ag^+^, PG fragmentation with the release of muramic acid in the medium	[140]
Commercial	5	Not indicated	Not indicated	No	MIC: 5; MBC: 20	AgNPs over pass cell wall and act on the cell membrane to damage the relative enzymes and interfere with cell metabolism. AgNPs enter bacteria cells and condensed DNA to prevent DNA from replicating and cells from reproducing. Simultaneously, AgNPs continuously act on the cell wall and cell membrane to destroy them	[141]
From *B. cereus* and glucose	32	Spherical	Not indicated	GEN, CHL	MIC and MEB were not indicated	Synergistic effect of AgNPs along with antibiotics in biofilm quenching, but the mechanism of action was not suggested	[142]
From *Streptomyces coelicolor* pigments by photo-irradiation within 20 min	28–50	Irregular	Not indicated	GEN, OXA	Only inhibition zones on solid cultures were measured. GEN: 14 mm; AgNPs + GEN: 22 mm; OXA: 10 mm; AgNPs + OXA: 20 mm	The synergistic activity of AgNPs with both antibiotics was attributed to the interaction of the AgNPs with hydroxyl and amide groups in the antibiotics	[143]
From *Alysicarpus monilifer* leaf extract	5–45 15 ± 2 (avg.)	Spherical deriving in nanoprisms	No indicated	No	MIC: 60; MBC: 80 (against coagulase-negative staphylococci): MIC: 80; MBC: 100 (against MRSA)	AgNPs are capable of affecting the integrity of cell membranes and interacting with disulfide bonds of intracellular enzymes, disturbing metabolic processes and inhibiting the major functions of bacterial cells, including cellular uptake and respiration	[144]
From chitosan solution	4–18, 6–8 (50% of the AgNPs)	Not indicated	Not indicated	No	AgNP-chitosan MIC: 10; MBC: 10 (*S. aureus*)	Chitosan-based AgNPs have a dual mechanism of action for antibacterial activity, the bactericidal effect of AgNPs, and the cationic effects of chitosan	[110]
From *Pseudomonas aeruginosa*	25–45	Spherical	Not indicated	No	MIC (pM): 0.4–3.2 (against MSSA –MRSA clinical strains). 3.2 (against *S. epidermidis*) MBC (pM): 0.8–3.2 (against MSSA -MRSA clinical strains) 6.2 (*S. epidermidis*)	Reference to previously reported mechanisms. Effect on the membrane: release of Ag^+^	[80]
Commercial	Not indicated	Not indicated	No	No	MIC: 100 (*S. aureus*)	Bacterial protein leakage by increasing the membrane permeability. Formation of ROS that inactivate LDH	[122]
Reduction with NaBH_4_ + Na citrate	4	Not indicated	Citrate/AMP	AMP linked to AgNP (AMP-AgNP)	MBC: 1 for AMP-AgNPs, MBC: 4 for AgNPs alone	No mechanism of action was proposed	[83]

Abbreviations: AMP: ampicillin; avg.: average; GEN: gentamicin; CHL: chloramphenicol; MBC: minimum bactericidal concentration; MIC: minimum inhibitory concentration; MIC_99_: minimum concentration that inhibits 99% of bacteria; MRSA: methicillin-resistant *Staphylococcus aureus*; MSSA: methicillin-sensitive *Staphylococcus aureus*; nM: nanomolar; OXA: oxacillin; PG: peptidoglycan.; pM: picomolar; STR: streptomycin.

## 7. Toxicity of AgNPs

A growing concern has emerged regarding the biological impacts of NP usage and possible risks to the environment and human health. NPs exhibit an exceptionally increased surface-to-volume ratio due to their ultra-small size. This provides reactivity and, hence, toxicity to these particles. Penetration of NPs across cell barriers is mostly size-dependent. Decreased size exponentially increases surface area resulting in higher levels of oxidation and DNA damaging capabilities [145]. Thus, this passes into living organism cells and can cause several cell lesions [146,147]. The toxicity of AgNPs has been reviewed [148] and it was concluded that cytotoxicity of AgNPs can be considered as dependent on different properties such as size, shape, dose, agglomeration, or aggregation; however, there are not presently adequate studies to obtain a concrete idea of the cytotoxicity of AgNPs or the mechanism behind the toxicity. Binding AgNPs with a coating layer of peptides or other suitable biocompatible molecules can lower toxicity at the time the antibacterial effectivity increases [137,149,150].

Reasonably, *in vitro* studies were performed before *in vivo* experiments. Although *in vitro* data is not a substitute for whole-animal studies, *in vitro* models can reveal toxicity mechanisms that can serve as a basis for further assessing the potential risk of NP exposure [151].

The mitochondrial function decreased significantly when the immortalized *in vitro* rat-liver derived cell line (BRL 3A) was used to evaluate the acute toxic effects of AgNPs (15–100 nm) at AgNP doses of 5–50 µg/mL [151]. Exposure of HT 1080 (human fibrosarcoma) and A431 cells (human skin/carcinoma) cells to AgNPs at doses up to 6.25 μg/mL caused apoptosis, oxidative stress, and morphology changes [152]. Concerns about the potential NP cytotoxicity and genotoxicity have increased in the last years leading to intensive studies [153,154,155]. *In vitro* experiments have evidenced that AgNPs are not only transported into cells and internalized, but also target endosomes and lysosomes [156,157], so that brain astrocytes accumulate AgNPs in a time-, dose-, and temperature-dependent way, likely involving endocytic pathways. AgNPs induce lung fibroblasts, impair the cellular membrane, and cause DNA damage and genotoxicity, chromosome aberration, and apoptosis [148,158,159,160]. Non-agglomerated AgNPs from the cell culture medium were detected as agglomerates within the human mesenchymal stem cells (hMSC) [146]. The silver agglomerates were located in the perinuclear region and the 80 nm AgNPs occurred mainly within endo-lysosomal structures, not in the cell nucleus, endoplasmic reticulum, or Golgi complex. Damage to hMSC vitality at concentrations of 10 µg/mL was evident when working on hMSC cultures doped with AgNPs (47 nm) [161]. ROS generation and oxidative stress play a crucial role in this context. AgNPs may induce geno- and cytotoxic effects in hMSC at high exposure concentrations although subtoxic levels may activate hMSC [161]. Exposure to AgNPs of human alveolar basal epithelial cells (A549) produced ROS generation and reductions in cell viability and mitochondrial membrane potential [153,162]. AgNPs showed more toxicity in A549 cells than in L132 normal human lung cells, which had no significant membrane leakage. Toxicity is dose-dependent and AgNPs target cancer cells rather than normal cells [162]. PVP-AgNPs do not have toxic effects on human pulmonary host cells (A549) at levels therapeutic to *A. baumannii* infection, being the IC_50_ = 130 µM [62]. Cytotoxicity was assessed according to ISO 10993-533 by monitoring the neutral red uptake assay using mouse fibroblasts NCTC 929, and tumor cells HeLa and HepG2 (100 μL; 1 × 10^5^ cells/mL) seeded into 96-well plates and left to adhere during 24 h [75]. Cells were exposed to 10-nm AgNPs previously dispersed and serially diluted at concentrations from 10.0 to 0.156 μg/mL. The bactericidal AgNP levels were non-cytotoxic in NCTC 929, HepG2, and HeLa tumor cells. Low toxicity to the cell lines HepG2 and HeLa was observed at 5.0 μg/mL while cytotoxicity was evident at 10 μg/mL [75]. AgNPs (20 nm) killed *S. aureus* but were non-toxic to HeLa cells [134]. Wypij et al. [97] have reported a high cytotoxic effect (IC_50_ about 4 μg/mL) of biogenic AgNPs (5–20 nm) *in vitro* using the mouse 3T3 fibroblasts and HeLa cell line. Cytotoxicity inconsistencies with results from previous research were possibly due to experimental differences [97]. The combined use of AgNPs and antibiotics has made it possible to reduce the dosage of both antimicrobials and their toxicity toward mouse fibroblasts and HeLa cells [97]. On the other hand, the AgNP concentration required for 50% reduction in viability in HeLa cells was 200 μg/mL using NPs of 45 nm size [163] or 100 μg/mL using NPs with a size of 62 nm [164]. Składanowski et al. [155] also reported AgNPs low cytotoxicity (IC_50_ = 64.5 μg/mL) against mouse fibroblasts (L929 cell line). The combination of some antibiotics with AgNPs at their MIC values decreased cell viability in comparison with untreated cells. The highest cytotoxic effect was detected for ampicillin/sulbactam, cefazolin, meropenem, and chloramphenicol combined with AgNPs, which was attributed to the additive cytotoxicity of the antibiotics and AgNPs [95].

Barbasz et al. [165] studied the toxicity of three AgNP types [uncapped (negatively charged), citrate-capped (negatively charged) and cysteamine-capped (positively charged)] with sizes of 11–14 nm towards histiocytic lymphoma (U-937) and human promyelocytic cells (HL-60) and found that uncapped negatively charged AgNPs exhibited the highest toxicity towards the tumor cell line. They concluded that the AgNP cytotoxicity mechanism is a combination of effects coming from the NP surface charge, released silver ions and the biological activity of stabilizing agent molecules, and that their results confirmed that disruption in mitochondrial functions and generation of oxidative stress are the main reasons of cell death.

Low doses of antibiotics and AgNPs slightly decrease cell viability (to 90–95%) in comparison with the control cells, depending on the antibiotics used [97], which suggests that AgNPs in combination with antibiotics have much potential for application as antimicrobial agents. AgNPs at concentrations ≤ 30 µg/mL did not display cytotoxic effects to human cells, blood, or to environmentally important organisms [166,167,168,169]. The results of extensive hemocompatibility tests based on plasma concentrations of activation markers, cell surface markers, and blood cell alterations reflected good hemocompatibility of AgNPs (12 nm) at concentrations up to 3 µg/mL. No biological relevant alterations during blood contact were observed but 30 µg AgNP/mL induced activation of various hematologic parameters and this dose should not be used *in vivo* [169].

An *in vivo* study on 60 healthy volunteers orally exposed to commercial AgNPs in a prospective, placebo-controlled, single-blind, dose-monitored, and cross-over design did not show clinically important changes in metabolic, urine, hematologic, physical findings, or imaging morphology after 14 days of exposure to 10 µg/mL (5–10 nm size) and 32 µg/mL (25–40 nm size) of AgNPs. Thus, exposure to low AgNP doses has no adverse or toxic effects on humans according to that study [170]. However, the results from different studies related to the toxic effects of AgNPs using experimental animals disagree and conclusions are controversial [124,171,172,173,174,175,176,177,178,179]. Generally, oral exposures to AgNP caused weight loss, inflammatory and immune responses, hepatic alterations, increased levels of neurotransmitters, and changed blood values in animal model experiments at concentrations of units or tens of mg/kg [171,172,173,174,176]. Kim et al. [106] found that 28 days of repeated oral doses of commercial AgNPs (60 nm) to Sprague–Dawley rats induced liver toxicity, affected coagulation of peripheral blood, and had a dose-dependent deposition of AgNPs in the blood, stomach, brain, liver, kidneys, lungs, and testes of the rats indicating that the AgNPs were systemically distributed in the tissues. A conclusion was that exposure to > 300 mg of AgNPs may result in only slight liver damage. A study on the oral toxicity of AgNPs (56 nm) over a 90-day period in F344 rats concluded that the target organ for the AgNPs was the liver in male and female rats [173]. A NOAEL (no observable adverse effect level) of 30 mg/kg of body weight/day and a LOAEL (lowest observable adverse effect level) of 125 mg/kg of body weight/day was suggested. Nevertheless, the LOAEL expressed by increased cytokine concentration was 0.5 mg/kg of body weight/day in mice following 28-day oral AgNP exposure [176]. Dermal toxicity studies showed that exposure to > 0.1 mg AgNPs/kg results in slight spleen, liver, and skin damage in guinea pigs, thus denoting that this administration way supposes more toxicity than oral or inhalation ways [180]. After subcutaneous injections of AgNPs at 62.8 mg/kg in rats, the NPs were translocated to the blood circulation and distributed to the kidney, liver, spleen, brain, and lung. Moreover, AgNPs caused blood–brain barrier destruction and neuronal degeneration [181,182]. However, doses of 62.8 mg/kg in the case of subcutaneous injection or 300 mg/kg in the case of oral administration are very high and they do not need to be administrated to treat bacterial infections, especially when AgNPs are combined with antibiotics [95].

To clarify the toxic effects of AgNPs, a genotoxicity test, oral and dermal toxicity test, skin toxicity test, and eye toxicity test was conducted according to the OECD test guidelines and GLP [178]. AgNPs (10 nm) did not show a severe toxic effect on microorganisms, mammalian cell lines, or target animal organs. Notwithstanding, certain concentrations of AgNPs induced cytotoxicity in microorganisms and mammalian cell lines. Abnormal signs or mortality following the acute oral or dermal exposure of rats at a dose of 2000 mg/kg were not found, making the LD_50_ of AgNPs for Sprague Dawley rats above 2000 mg/kg. Other authors agree with these results [183]. In the dermal irritation and corrosion test, the AgNPs did not generate abnormal clinical signs or mortality in New Zealand White Rabbits and did not induce any erythema, eschar, or edema formation during the experimental period. In the skin sensitization test, a weak skin sensitization effect was found in one guinea pig (5%), which showed discrete or patchy erythema induced by AgNPs [178].

Changes in the acute toxicity of intraperitoneally administered AgNPs (10, 60 and 100 nm) in BALB/c mice (0.2 mg/mouse) have been observed [184]. The smaller AgNPs exhibited more toxicity than the larger ones. After 6 h of administration congestion, vacuolation, single cell necrosis, focal necrosis in the liver, congestion in the spleen and apoptosis in the thymus cortex was observed. These results agree with other studies on the acute toxicity of citrate-capped and PVP-capped AgNPs in mice but using intravenous injection (a single dose of 10 mg/kg). After 24 h, the highest silver concentrations occurred in the spleen and liver, followed by the lung, kidney, and brain [185].

Unfortunately, the issue of the relevant AgNP dose required for system or local elimination of infection is not addressed yet. Similarly, pharmacological and pharmacokinetic data on AgNPs have not been described so far. Therefore, prediction of the therapeutic AgNP doses and their adverse effects is very difficult at this time. This is still an open field, which requires further exploration to determine if AgNPs combined with antibiotics may be effective for the local and systematic therapy of infectious diseases without showing adverse effects.

Concerning toxicity in humans, there is little information on assays performed in volunteer patients [170,186]. NPs with size ≤ 35 nm can penetrate and cross the blood–brain barrier, particles with size ≤ 40 nm may enter nuclei of cells, and those with size 1–100 nm can cross the biological membrane and can be translocated inside cell organelles or entities such as the mitochondria, lysosome, nucleus, and others [187]. AgNPs can induce size-dependent cytotoxicity in human lung cells due to the substantial release of Ag in the cell [25]. Silver-coated wound dressing tested in human burns patients produced reversible hepatotoxicity and argyria-like discoloration of the treated area of skin, elevated plasma and urine silver concentrations, and increased liver enzymes. AgNP doses in the 5–10 μg/mL range proved toxic in eukaryotic cells. If effective antimicrobial AgNP doses were higher than cytotoxic levels, its practical use in humans would be problematic [188]. The pharmacokinetic and pharmacodynamic characteristics of the NPs, including AgNPs, have been evaluated but in-depth knowledge is needed [189]. The key issue to overcoming toxicity problems in AgNP treatments is finding silver nanocomposites capped with antibiotics that can act as efficient antimicrobial systems against antibiotic-resistant bacteria without toxicity to human tissues. The objective must be optimizing the ratio of maximal antibacterial activity/null or minimal toxicity to human organs. Thus, studies aimed at standardizing the optimal size, shape, purity, stability, capping agents, antibiotic combination, and doses of AgNPs to permit clinical usage on critically infected patients with minimal or null side effects are needed.

## 8. Conclusions and Future Perspectives

The abuse of antibiotics worldwide has contributed to the development of MDR infectious bacteria. This is a real problem for public health because nosocomial infections are very difficult to cure. There is an urgent demand for new treatments to counteract the increased morbidity/mortality rates and treatment costs. Nanotechnology has become a new tool to fight against MDR microorganisms. Metal NPs may be used in medicine to combat the infections caused by these bacteria. Particularly, AgNPs have been applied to this objective and have been shown to be very effective against *A. baumannii, P. aeruginosa*, Enterobacteriaceae, and MDR *S. aureus*. The mechanism of action of AgNPs involves interactions at various levels. They change the plasma membrane permeability causing the release of the intracellular content and leading to cell death. They stop DNA replication, inhibit the expression of ribosomal subunits and inactivate proteins/enzymes. They change the normal function of membrane-bound respiratory enzymes and lead to the formation of ROS with oxidative deterioration of cell content. AgNP toxicity is dependent on dose, size, shape, and capping/coating agents among other factors. The addition of suitable antibiotics increases the antibacterial activity and decreases toxicity due to synergistic effects. Low sizes (5–30 nm) are more effective against bacteria than larger diameters. The shape and the electrical charge influence its activity. AgNP toxicity to animal cells has been studied mainly *in vitro* on cell cultures but also *in vivo*. The smallest AgNPs display the highest toxicity to animal cells while large sizes are less toxic. Therefore, the use of small AgNPs must be exerted with great care. Different researchers do not always agree on toxicological aspects. The administration way influences the toxicity in animals. The doses should be as low as possible but the relevant dose required for the elimination of infection is not addressed. Thus, more studies are needed to establish the best compromise between toxicity and therapeutic effects by finding the best balance encompassing dose/size/shape/charge and coating with substances such as suitable antibiotics and/or capping compounds that can improve AgNP effectiveness. Detoxification of affected organs is a very important issue to be addressed. Another perspective to be considered in future is the study of possible bacterial mechanisms of resistance against NPs. Owing to the urgent action demanded by the WHO against MDR bacteria such research is of paramount importance. The answer to the research question of this review is yes, AgNPs can be useful in treatments but more research (mainly in toxicology) is needed before AgNP-based therapies may be approved for use in clinical medicine by the Food and Drug Administration or the European Medicine Agency.

## Data Availability

Not applicable.
